# Conscious sedation in Spanish dental schools: Current situation

**DOI:** 10.1002/cre2.190

**Published:** 2019-05-09

**Authors:** María Inmaculada Vela, María Arregui, Lluis Giner, Esther Jiménez

**Affiliations:** ^1^ Dental School International University of Catalonia (Universidad Internacional de Catalunya) Barcelona Spain; ^2^ Education School International University of Catalonia (Universidad Internacional de Catalunya) Barcelona Spain

**Keywords:** competency, conscious sedation, curriculum, degree in dentistry

## Abstract

The current Spanish curricula for degrees in dentistry include conscious sedation (CS) as a basic training competency. However, is the CS training delivered by Spanish dental schools a consensus‐based educational framework enabling students to use this anesthetic technique after graduation? To answer this research question, a study was designed aiming to identify the strategies used to teach this competency in Spanish dental schools and the characteristics of teaching. The authors reviewed legislation concerning officially established requirements for a degree in dentistry as well as curricula currently taught in Spain. Our analysis identified clear discrepancies among the schools of dentistry studied. The only overlap was observed in reference to the level of proficiency imparted, which prevents Spanish dentistry students from using this anesthetic technique after graduation. Specific features of the normative framework and of the Spanish legislative system underlying the design of the present curricula of degrees in dentistry would explain the discrepancies in CS competencies taught at our schools of dentistry. Almost 10 years since its implementation and in light of the new demands of the complex society in which we live, Spanish universities must unify their educational criteria regarding CS training to ensure the appropriate qualification of our new dentists in this technique.

## 1 INTRODUCTION

After the Bologna Declaration (1999) and the establishment of the European Higher Education Area (EHEA), the National Agency for Quality Assessment and Accreditation (Agencia Nacional de Evaluación de la Calidad y Acreditación—ANECA) commissioned a network of public and private universities to design a new dentistry curriculum, which was published under the title “Libro Blanco del Título de Grado en Odontología [White paper on the degree in dentistry]” (ANECA, 2004).

Prior to publication, this network developed a questionnaire to assess the competencies taught by the current curricula at that time (Real Decreto 970/1986, 1986), highlighting one competency for its overall low score: “having knowledge of conscious sedation techniques applied to dental treatments.”

The low score of this competency was then attributed to the fact that conscious sedation was “a technique (the technique of conscious sedation) still not well established and used only in special cases …” (Chapters 9 and 10. Documentation of proficiency assessment and the comparison between proficiency and academic and professional experience. Analysis of results. p. 118; ANECA, 2004).

Over the years, persistent prevalence of fear and dental anxiety, as well as variations in social demands and demographic changes, have promoted a new training profile in which the acquisition of this skill is highly important for new Spanish dental graduates.

As published studies clearly show, these are international factors compromising the oral health of the world population that are also shared by Spain; thus, there is a need to develop educational training programs to meet the needs of society (Abdulwahab et al., 2010; Chanpong, Haas, & Locker, 2005; Facco & Zanette, 2017).

Indeed, despite advances in dental techniques and technologies, the latest Spanish national survey on oral and dental health (2015; Consejo General de Colegios de Dentistas de España, 2016b) reported that fear is the third leading reason why Spaniards avoid visiting the dentist. This figure has increased by 3% since the 2010 survey. Furthermore, studies evaluated by the General Dental Council of Spain (Consejo General de Dentistas de España—CGDE) highlight that 65% of dental patients suffer from severe dental phobia (CGDE, 2015).

Variations in social demands and demographic changes as Spain transitions into an aging society must be analyzed. Europe has experienced a significant increase in the number of people older than 65 years (Statistical Office of the European Communities [Eurostat], 2015). This demographic group is particularly noticeable in Spain, accounting for 18.7% of the population in 2016 and may account for up to 25.2% in 2031 (Instituto Nacional de Estadística, 2018), as Spain has the European region's highest life expectancy rates (Statistical Office of the European Communities [Eurostat], 2018).

From the dentist's point of view, the management of elderly patients is a considerable challenge because the members of this demographic group age differently. The healthy elderly ask to be treated with the latest surgical and restorative techniques to recover their image and oral function, but their decreased functional reserve, which is typical of aging, lowers their ability to adapt to stressful situations, including the use of these techniques (Caballero & Caballero, 1998). Elderly patients with physical and cognitive deterioration, who show increased vulnerability and decreased ability to control their environment, require individualized treatment plans with health interventions under conscious sedation (CS; Bermejo, 2015).

In summary, Spain shares a series of international factors that seriously affect the oral health of its population and require consensus‐based training in CS to enable recent graduates to use this technique safely, a task not exempt from difficulties according to the universities of different European and non‐European countries who share our concerns, including the United Kingdom and Ireland (Leitch & Jauhar, 2006), the United States (Boynes, Lemak, & Close, 2006), Australia (Moore, Boynes, Cuddy, Giovannitti, & Zovko, 2009), Japan (Morse, Sano, Fujii, & Kanri, 2004), and Jordan (Al‐Shayyab, Ryalat, Dar‐odeh, & Alsoleihat, 2013).

The following question emerges from the abovementioned research findings: Is the CS training delivered by Spanish dental schools a consensus‐based educational framework enabling students to use this anesthetic technique after graduation?

To answer this question, in October 2015 at the School of Dentistry at International University of Catalonia (Facultad de Odontología de la Universidad Internacional de Cataluña, Barcelona), we started to identify different strategies adopted at Spanish schools of dentistry to analyze CS competency in new curricula currently in use for degrees in dentistry.

## 2 MATERIALS AND METHODS

This study was divided into two phases.

### 2.1 First phase

We reviewed the Libro Blanco del Título de Grado en Odontología [White paper on the degree in dentistry] (ANECA, 2004) and the Orden Consejo Interuniversitario Nacional (CIN) [National Inter‐university Council Order]/2136/2008 (Orden CIN/2136/2008, 2008) issued by the Ministry of Science and Innovation (Ministerio de Ciencia e Innovación), which regulate the minimum teaching requirements of the degree in dentistry because they are the main sources on which the schools of dentistry base the design of their current curricula. Our objective was to gather information regarding the description of CS competency in both documents.

The first document we reviewed, the Libro Blanco ANECA (ANECA, 2004), defines the common mandatory contents of the degrees in dentistry into seven training modules. In three, CS is included in the specific basic and professional training competencies. They define proficiency in CS as “having knowledge on conscious sedation techniques applied to dental treatments” and only explain the content as “conscious sedation in dentistry,” with no additional information (Estructura general de la titulación. Contenidos comunes obligatorios [General structure of the degree. Mandatory common contents], pp. 123–146; ANECA, 2004).

The second document, the Orden CIN [National Inter‐university Council Order]/2136/2008 (Orden CIN/2136/2008, 2008), establishes that through the Patología y Terapéutica Médica y Quirúrgica General [Medical pathology and therapeutics and general surgery] module, dentists have to “acquire knowledge on the pharmacological bases of different anaesthetic techniques, both local and general, as well as on the role of sedation and general anaesthesia in the management of the dental patient” (Annex I, Section 5. Planificación de las enseñanzas [Teaching plans], p. 31691; Orden CIN/2136/2008, 2008).

### 2.2 Second phase

We started the second phase by accessing the website of the Ministry of Education, Culture and Sports (Ministerio de Educación, Cultura y Deporte; Ministerio de Educación, Cultura y Deporte, 2016) to identify all academic centers authorized to teach the degree of dentistry in Spain, finding 23 universities authorized for the 2017–2018 academic year (Table 1).

**Table 1:** List of Spanish centers offering a degree in dentistry (2017–2018 academic year)

University	School	Online link for access to the curriculum
1. University of Valencia [Universitat de València, Estudi General—UV]	School of Medicine and Dentistry [Facultad de Medicina y Odontología]	http://www.uv.es/uvweb/universidad/es/estudios‐grado/oferta‐grados/oferta‐grados/grado‐odontologia
2. University of Seville [Universidad de Sevilla—US]	School of Dentistry [Facultad de Odontología]	http://www.us.es/estudios/grados/plan_173?p=7
3. University of Murcia [Universidad de Murcia—UM]	School of Medicine [Facultad de Medicina]	http://www.um.es/web/medicina/contenido/estudios/grados/odontologia
4. University of Granada [Universidad de Granada—UGR]	School of Dentistry [Facultad de Odontología]	http://grados.ugr.es/odontologia/pages/infoacademica/estudios#_doku_guias_docentes
5. University of Salamanca [Universidad de Salamanca—USAL]	School of Medicine [Facultad de Medicina]	http://www.usal.es/node/4486/plan_estudios
6. University of Santiago de Compostela [Universidad de Santiago de Compostela—USC]	School of Medicine and Dentistry [Facultad de Medicina y Odontología]	http://www.usc.es/graos/gl/graos/ciencias‐saude/odontoloxia
7. Complutense University of Madrid [Universidad Complutense de Madrid—UCM]	School of Dentistry [Facultad de Odontología]	http://odontologia.ucm.es/grado
8. King Juan Carlos University [Universidad Rey Juan Carlos, Madrid—URJC]	School of Health Sciences [Facultad de Ciencias de la Salud]	https://miportal.urjc.es/guiasdocentes/mostrarGuias.jsp
9. University of the Basque Country [Euskal Herriko Unibertsitatea—EHU]	School of Medicine and Nursing [Facultad de Medicina y Enfermería]	http://www.ehu.eus/es/web/medikuntza‐odontologia/odontologiako‐gradua‐2015‐2016
10. University of Zaragoza [Universidad de Zaragoza—UNIZAR]	School of Health Sciences and Sports [Facultad de Ciencias de la Salud y del Deporte]	http://titulaciones.unizar.es/odontologia/planesestudio.
11. University of Oviedo [Universidade de Oviedo—UNIOVI]	School of Medicine and Health Sciences [Facultad de Medicina y Ciencias de la Salud]	http://sies.uniovi.es/ofe‐pod‐jsf/web/oferta/seccion‐1.faces
12. Alfonso X El Sabio University [Universidad Alfonso X El Sabio, Madrid—UAX]	School of Health Sciences [Facultad de Ciencias de la Salud]	http://www.uax.es/grado‐en‐odontologia.html
13. CEU Cardenal Herrera University [Universidad CEU Cardenal Herrera, Valencia—UCHCEU]	School of Health Sciences [Facultad de Ciencias de la Salud]	https://www.uchceu.es/estudios/grado/odontologia
14. Valencia Catholic University Saint Vincent Martyr [Universidad Católica de Valencia San Vicente Mártir—UCV]	School of Medicine and Dentistry [Facultad de Medicina y Odontología]	https://www.ucv.es/oferta‐academica/grados/grado‐en‐odontologia/seccion/13
15. Catholic University of Murcia [Universidad Católica San Antonio de Murcia—UCAM]	School of Health Sciences [Facultad de Ciencias de la Salud]	http://www.ucam.edu/estudios/grados/odontologia‐presencial/plan‐de‐estudios
16. University of Barcelona [Universitat de Barcelona—UB]	School of Medicine and Health Sciences [Facultad de Medicina y Ciencias de la Salud]	http://www.ub.edu/web/ub/es/universitat/campus_fac_dep/facultats_escoles/facultat_odontologia/facultat_odontologia.html
17. European University of Madrid, Villaviciosa and Alcobendas Campus [Universidad Europea de Madrid, Campus Villaviciosa y Alcobendas—UEM]	Facultad de Ciencias Biomédicas y de la Salud	http://madrid.universidadeuropea.es/estudios‐universitarios/estudiar‐odontologia
18. European University of Madrid, Valencia Campus [Universidade Europea de Madrid, Campus Valencia—UEM]	Centro de Educación Superior Valencia	http://madrid.universidadeuropea.es/estudios‐universitarios/estudiar‐odontologia
19. European University of Valencia [Universidad Europea de Valencia—UEV]	School of Health Sciences [Facultad de Ciencias de la Salud]	http://valencia.universidadeuropea.es/estudios‐universitarios/estudiarodontologia/grados.
20. Miguel de Cervantes European University [Universidad Europea Miguel de Cervantes, Valladolid—UEMC]	School of Health Sciences [Facultad de Ciencias de la Salud]	https://www.uemc.es/grados/grado‐en‐odontologia/plan
21. CEU San Pablo University [Universidad San Pablo‐CEU, Madrid—USPCEU]	School of Medicine [Facultad de Medicina]	http://www.uspceu.com/oferta‐academica/grado/grado‐en‐odontologia
22. University of the Balearic Islands [Universitat de les Illes Balears—UIB]	ADEMA University College of Dentistry [Escuela Universitaria de Odontología ADEMA]	http://estudis.uib.es/es/grau/odontologia/GODO‐P/assignatures.html
23. International University of Catalonia [Universitat Internacional de Catalunya—UIC]	School of Dentistry [Facultad de Odontología]	http://www.uic.es/es/odontologia/grado‐odontologia/plan‐de‐estudios

We were able to access the curricula and teaching guides of 20 schools of dentistry from their webpages. Therefore, the two schools with no information available online were excluded from the study, as was our own.

## 3 RESULTS

All curricula of the 20 schools of dentistry analyzed include CS competency, albeit using different strategies.

We use the term “strategy” considering its meaning of “technique and set of activities aimed at achieving an objective,” as recognized by the Spanish Royal Academy of Language.

On the basis of this definition, we will divide the presentation of the results of our study into two sections:

- The “technique” used: In our study, this would correspond to the way in which competence in CS is introduced.
- Achievement of the objective: in our case, training and enabling students to use CS.

### 3.1 Strategy: The technique used

In the 2017–2018 academic year, the schools introduced the competence through 23 subjects that, according to the educational legislation, should belong to the General Medical and Surgical Pathology and Therapeutics [Patología y Terapéutica Médica y Quirúrgica General] module (Orden CIN/2136/2008, 2008). However, only 52% related to this module (Table 2).

**Table 2:** Courses grouped by content blocks (ANECA White Paper) according to their thematic scope and their taxonomic level

Block 4: pathology and general medical and surgical therapeutics
Surgical pathology
Surgical pathology I
Surgical pathology and reanimation
Pharmacology, anesthesia, and reanimation
Anesthesia and reanimation
Anesthesiology
Clinical pharmacology, human nutrition, anesthesia, and reanimation
Pharmacology
Fundamentals of surgery, anesthesia, and reanimation
General and clinical pharmacology
Surgical pathology II
Maxillofacial surgery
Block 5: pathology and general medical and surgical therapeutics in dentistry	
Applied surgical pathology	
Surgical pathology applied to dentistry	
Oral surgical pathology	
Mouth surgery	
Oral surgery	
Mouth surgery and implantology	
Mouth surgery (I)	
Advanced surgical techniques	
Block 6: pathology and restorative and rehabilitative therapeutics in dentistry	
Dentistry in special patients	
Gerontology and treatment of special patients	
Orofacial pain (elective)	

Abbreviation: ANECA, Agencia Nacional de Evaluación de la Calidad y Acreditación.

**Figure 1:**
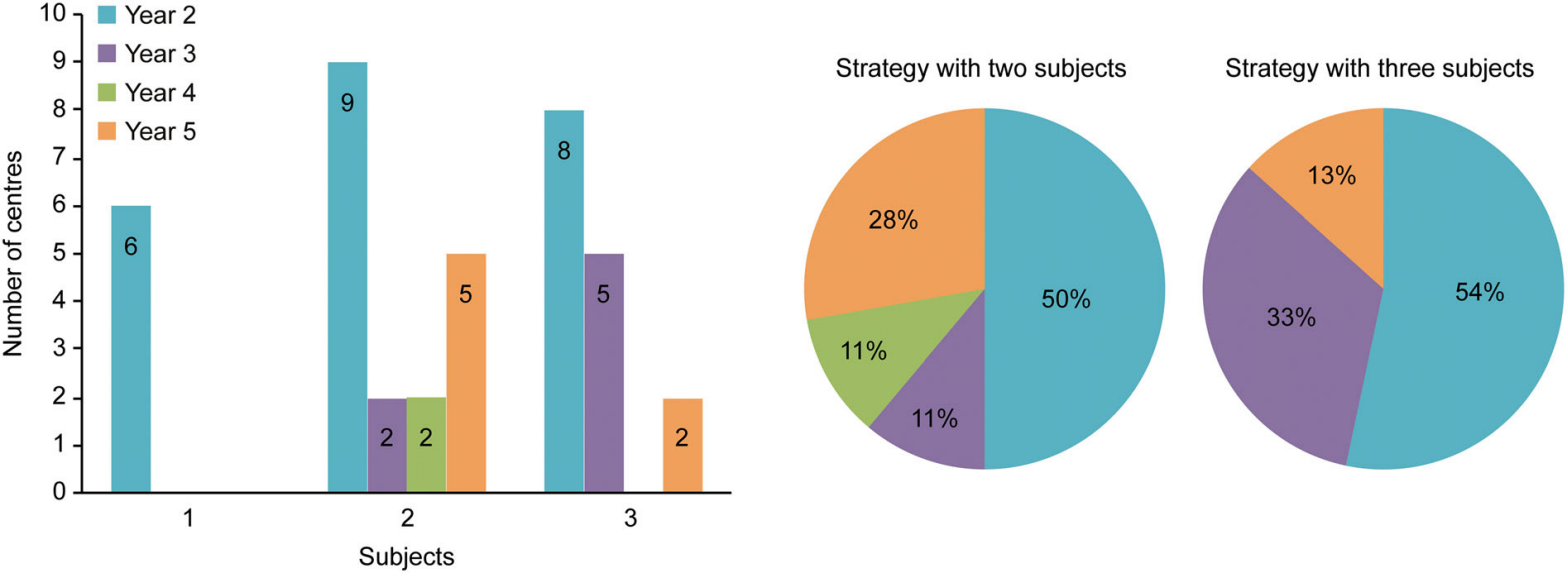
Strategy for the introduction of conscious sedation competency. Distribution of subjects by year of the degree program

We found that each school allocates a different number of subjects: Most schools use two (45%) subjects, with some using one (30%) or three (25%) subjects (Table 3 and Figure 1).

**Table 3:** Strategy used by Spanish schools of dentistry to introduce conscious sedation competency

University	Subject	Year of the degree program
1 subject		
K.	Anesthesiology	2nd
O.	Clinical pharmacology, human nutrition, anesthesia, and reanimation	2nd
P.	Clinical pharmacology, human nutrition, anesthesia, and reanimation	2nd
Q.	Clinical pharmacology, human nutrition, anesthesia, and reanimation	2nd
R.	Anesthesia and reanimation	2nd
S.	Pharmacology, anesthesia, and reanimation	2nd
2 subjects		
A.	Surgical pathology	2nd
	Dentistry in special patients	5th
F.	Surgical pathology applied to dentistry	2nd
	Pharmacology	2nd
G.	Surgical pharmacology, anesthesia, and reanimation	2nd
	Oral surgery	3rd
I.	Anesthesia and reanimation	2nd
	Maxillofacial surgery	5th
J.	Oral surgical pathology	3rd
	Pharmacology	2nd
N.	Dentistry in special patients	5th
	Applied surgical pathology	2nd
T.	Anesthesia and reanimation	2nd
	Advanced surgical techniques	5th
D.	Orofacial pain	4th
	Gerontology and treatment of special patients	5th
H.	Anesthesia and reanimation	2nd
	Orofacial pain	4th
3 subjects		
B.	Surgical pathology I	2nd
	Surgical pathology II	3rd
	Oral surgery and implantology	3rd
C.	Pharmacology, anesthesia, and reanimation	2nd
	Oral surgery	3rd
	Dentistry in special patients	5th
E.	Surgical pathology II	2nd
	General and clinical pharmacology	3rd
	Oral surgery I	3rd
L.	Pharmacology	2nd
	Surgical pathology and reanimation	2nd
	Dentistry in special patients	5th
M.	Pharmacology	2nd
	Surgical pathology	2nd
	Oral surgical pathology	2nd

If we relate this fact with the academic year in which the subjects are taught, approximately 60% of the 23 subjects the schools allocate to introduce competence in CS correspond to the second year of pursuing the degree; when the centers teach only one subject, it is always part of the second‐year curriculum. In cases where two or three subjects are used to deliver the content, the percentage of second‐year subjects is higher than that of the other courses of the degree (Table 3 and Figure 1).

Importantly, in presenting the curricula analysis results, we replaced the names of the schools of dentistry included in the present study with uppercase letters for confidentiality purposes in several of the tables prepared.

### 3.2 Strategy: Achievement of an objective

To examine student training and whether it enables students to use CS, we analyzed the subjects from various perspectives:

1. Title of the subject. Several schools share the same subject titles, although the topics addressed vary on the basis of the center in which they are taught. Therefore, they are considered different subjects.
2. European Credit Transfer System (ECTS) credits assigned. The ECTS is the system adopted by all universities of the EHEA to standardize their studies. Each ECTS credit requires 25 to 30 hr of education, including lecture time, study time, tutoring, seminars, assignments, rotations or projects, and time required to prepare and to perform examinations and evaluations.

The data obtained reflect that most subjects—almost 60%—that include CS among their competencies assign six ECTS credits (from 150 to 180 hr). However, the syllabus only allocates one topic for CS training, presented as a lecture (from 1 to 2 hr).

- Semester in which they are taught. Most subjects on CS competency are taught for a single semester (64%), subject to the degree and semester in which they are taught.
- Definition of the level of proficiency. From the data extracted from the teaching guides, only 51% of subjects reflect a level of proficiency to be acquired consistent with the description of the content on CS.

The level of proficiency is not uniformly described. Several formats (Table 4) are used. We considered that they all reflect Level 2 of specific proficiencies according to the ANECA White Paper (ANECA, 2004).

**Table 4:** Types of formats used to describe the level of competency and the number of courses in which they are taught

Format	Description	Number of courses
1	Acquiring knowledge of anesthesiology and resuscitation (including local, regional, and general anesthesia and sedation)	2
2	Having knowledge of the pharmacological bases of different local and general anesthetic techniques, as well as the role of sedation and general anesthesia in managing dental patients	16
3	Knowing pharmacological principles, applied anatomy, and different clinical techniques for local, regional, and general oral anesthesia	1
4	Knowing the basics of local or general anesthesia and sedation in the dental patient	1
5	Knowing the sedation techniques used in dentistry, understanding the principles of sedation in dentistry and knowing the techniques used in the dental clinic, and knowing and understanding conscious sedation	1
6	Assessing techniques of sedation and general anesthesia in dentistry, as well as underlying risk factors	1
7	Being competent in the indication, planning and prevention, and resolution of risks of an anesthetic or sedative procedure and knowing the medicines, doses, and side effects	1
8	Knowing special dental treatment techniques: general anesthesia and sedation	1
9	Being able to describe and explain the anesthetic techniques used in oral surgery	1

Having knowledge on: dentists must have a deep theoretical knowledge on and understanding of the subject, although they only need limited clinical or practical experience because they are not expected to solve the clinical problem independently or because this knowledge is necessary for other disciplines.

- Description of the contents of proficiency in CS. In this analysis, wide variability was observed that affected subjects with the same title but were taught in different centers, such as the subjects “Anestesia y Reanimación” [Anesthesia and resuscitation] and “Odontología en Pacientes Especiales” [Dentistry in special patients], taught at four different schools (Table 5).

**Table 5:** Subject “anesthesia and resuscitation”: Analysis of differences by center in which the competencies are taught

University	Credits	Training activities
R	3	Total hours of the subject: 90
		Face‐to‐face hours
		Theoretical lessons: 21
		Practical lessons: 12
		Non‐face‐to‐face hours
		Autonomous student work: 57
T	3	Total hours of the subject: 101
		Face‐to‐face hours (1‐hr sessions)
		Theoretical lessons: 25
		Practical lessons: 16
		Evaluation: 4
		Non‐face‐to‐face hours
		Autonomous student work: 56
H	4	Total hours of the subject: 100
		Face‐to‐face hours: 50
		Theoretical lessons: 36
		Practical lessons: 4
		Evaluation: 10
		Non‐face‐to‐face hours
		Autonomous student work: 44
I	3	Total hours of the subject: 75
		Face‐to‐face hours: 50
		Theoretical lessons (?)
		Practical lessons (?)
		Non‐face‐to‐face hours
		Autonomous student work: 25
University	Credits	Contents on sedation
R	3	Inducer pharmacology, medicine inhalation, muscle relaxants, sedation in the dental office, and types of sedation
T	3	T10: sedation in odontostomatology
H	4	Module IV: sedation. Topic 6: sedation in dentistry and complications Module V: monitoring. Topic 10: monitoring in anesthesiology
I	3	Module IV: sedation 6. Sedation in dentistry and complications Outpatient anesthesia 10. Outpatient anesthesia, preanesthetic evaluation, anesthetic considerations, appropriate procedures for outpatient anesthesia, immediate postoperative period, and outpatient pediatric anesthesia

The results suggest that although the curricula of the dental degree include the general objectives and the learning content to train students in pain control, the schools have not yet introduced a formal training program in CS enabling students to use this anesthetic technique after graduation.

## 4 DISCUSSION

This study aims to review the strategies used in our schools of dentistry to introduce competence in CS and was conducted to answer a specific research question: Is the CS training delivered by Spanish dental schools a consensus‐based educational framework enabling students to use this anesthetic technique after graduation?

The ambiguity of state requirements (Real Decreto 970/1986, 1986)—not detailing the level of sedation in which future dentists are to be trained—and the imprecision of the educational framework (ANECA, 2004) created a dilemma within the dental curriculum regarding the adequate amount and content of CS training for the dental student. Considering the flexibility provided by the Spanish legal system to universities, it is not surprising that the defining characteristic of the results of our study is disagreement among schools.

The first part of our research question is thus answered: In Spain, the schools of dentistry do not offer consensus‐based training for competence in CS.

Another cause for this failure is that the guidelines recommended by professional associations, such as the CGDE, for the teaching and practice of CS (Consejo General de Colegios de Dentistas de España, n.d.), based on American Dental Association (2007) guidelines, arrived too late (2008), when the curricula had already received ministerial approval for their implementation in the academic year 2009–2010.

In this document, they suggested a minimum of 14 hr of training to achieve proficiency in this technique.

The analysis of the curricula and teaching guides in the academic year 2017–2018 showed that training hours are insufficient and are far from the minimum of 14 hr recommended by the CGDE according to American Dental Association guidelines (Consejo General de Colegios de Dentistas de España, n.d.; American Dental Association, 2007). Although CS, in most cases, is among the subject competences, with an average of six ECTS credits of teaching load (60% of the subjects), CS training is limited to a single lecture usually lasting from 1 to 2 hr. Even when the schools allocate three subjects to provide this instruction (only 25% of the schools analyzed), the hours are insufficient.

From our point of view, this little time would not meet the goal of “acquiring a broad theoretical knowledge …” that corresponds to the level of competence Spanish dental schools consider a mandatory requirement, the only common ground among them.

Therefore, answering the second part of our research question, the degree of dentistry in Spain is limited to providing a pharmacological basis for CS and knowledge of its effects on patients, not enabling dentists to use CS techniques.

## 5 CONCLUSIONS

Our degrees in dentistry should meet the guidelines of the “Profile and Competencies for the European Dentist” (Plasschaert, Holbrook, Delap, Martinez, & Walmsley, 2007), which establish that dentists must competently identify, evaluate, and treat anxiety with both pharmacological and behavioral techniques.

CS is a basic pharmacological technique to manage dental fear and anxiety. Similar to other European countries, the challenges posed by the increase in dental phobia and population aging create an urgent need to allow students to master this competence.

The results of our study show there is no regulated CS teaching, and thus, the degree in dentistry does not enable our dentists to use this technique (Consejo General de Colegios de Dentistas de España, 2016a).

As Botelho, Oancea, Thomas, Paganelli, and Ferrillo (2017) state, “a shared problem is a problem reduced by half.” Therefore, future studies could complement the data we have collected with a strengths, weaknesses, opportunities, and threats survey sent to each Spanish dental school regarding CS training and their proposals to develop a training program.

Universities, similar to any organization, can improve their processes and results by incorporating new techniques to meet social needs and demands.

To advance in this area, one possibility would be to design new academic training programs on the subject of competence in CS, mainly involving stakeholders such as professional associations, dentists, and anesthesiologists. This cooperation would provide an educational response to current demands, helping to eliminate the reluctance of other groups to the use of sedation techniques by dentists (Consejo General de Colegios de Dentistas de España, 2016a), a circumstance not unique to our country (Costa, Valadao, & Costa, 2010; Shearer, Wilson, & Girdler, 2004).

However, given our membership in the EHEA, another possibility would be for universities to develop their own programs by consensus, taking as reference the European countries that have included CS in the degree in dentistry since the end of the last century (Leitch & Girdler, 2000).
